# MHY4571, a novel diarylcyclohexanone derivative, exerts anti-cancer activity by regulating the PKA-cAMP-response element-binding protein pathway in squamous cell lung cancer

**DOI:** 10.1186/s40164-022-00324-8

**Published:** 2022-10-08

**Authors:** Jae Heun Chung, Ho Jung Choi, Yong Jung Kang, Yun Seong Kim, Sang-Yull Lee, Ryuk Jun Kwon, Han-Sol Jeong, Su-Jung Park, Yeongmu Jeong, Dongwan Kang, Jeongin Ko, SangGyun Noh, Hae Young Chung, Hyung Ryong Moon, Seong Hoon Yoon

**Affiliations:** 1 Department of Internal Medicine, Pusan National University, Pusan National University Yangsan Hospital, Beomeo-ri, Mulgeum-eup, Yangsan, 50612 South Korea; 2 Department of Biochemistry, School of Medicine, Pusan National University, Beomeo-ri, Mulgeum-eup, Yangsan, 50612 Republic of Korea; 3 Department of Family Medicine, Pusan National University, Pusan National University Yangsan Hospital, Yangsan, 50612 Republic of Korea; 4 School of Korean Medicine, Pusan National University, Yangsan, 50612 Republic of Korea; 5 Department of Manufacturing Pharmacy, College of Pharmacy, Pusan National University, Geumjeong-gu, Busan, 46241 Republic of Korea; 6 Research Institute for Convergence of Biomedical Science and Technology, Pusan National University Yangsan Hospital, Yangsan, 50612 Republic of Korea

**Keywords:** Lung cancer, MHY4571, Carcinogenesis, Cyclic AMP-Dependent Protein Kinases, cAMP response element-binding protein

## Abstract

**Background:**

The protein kinase A (PKA)/cAMP response element-binding protein (CREB) has been suggested to be related to the inhibition of the proliferation of non-small cell lung cancer (NSCLC) cells. This study aimed to investigate the efficacy of a novel diarylcyclohexanone derivative, MHY4571, in regulating the PKA-CREB pathway and to study its anti-tumor role in squamous NSCLC.

**Methods:**

We designed MHY4571 as a novel PKA inhibitor with acceptable in silico ADME properties and tested it in vitro in lung cancer cell lines and in vivo in xenograft and orthotopic mouse models of squamous cell lung carcinoma.

**Results:**

MHY4571 inhibited PKA activity (> 70% inhibition) and suppressed the expression of p-PKA and p-CREB dose-dependently. MHY4571 treatment reduced lung cancer cell viability and promoted caspase 3-dependent apoptotic cell death. Orally administered MHY4571 significantly suppressed lung tumor growth in xenograft and orthotopic mouse models. PKA catalytic subunit alpha-silencing by siRNA (siPKA) strongly attenuated CREB phosphorylation; siCREB did not alter PKA protein levels or its phosphorylation, suggesting that PKA is an upstream regulator of CREB activity. MHY4571 acted synergistically with cisplatin (on co-treatment) to induce apoptotic cell death in lung cancer cells.

**Conclusions:**

Our results imply that MHY4571 may be a potential drug candidate for squamous cell lung cancer treatment.

**Supplementary Information:**

The online version contains supplementary material available at 10.1186/s40164-022-00324-8.

## Background

The development of targeted therapies has redefined the treatment paradigm for patients with non-small cell lung cancer (NSCLC) harboring actionable driver mutations [1]. However, a paucity of actionable driver mutations and the emergence of acquired resistance are unmet needs for researchers. Overall, 80–85% of all human lung cancers are NSCLCs, which comprise two major histological subtypes: adenocarcinoma and squamous cell carcinoma. Targetable oncogenes have not been identified in the squamous cell carcinoma histological subtype of NSCLC; in contrast, several such oncogenes have been found in the adenocarcinoma subtype. Consequently, the five-year survival rates among patients with advanced squamous NSCLC treated with current chemotherapeutic regimens are approximately 5–6% [2, 3]. Advances in systemic therapies using immunotherapy have recently shown promise in the clinic [4, 5]. Although encouraging, the benefits of immunotherapy are still restricted to a small proportion of lung cancer patients, and progression occurs within a few months of administering the therapy. Therefore, target discovery and mechanism-of-action examinations have essential roles in drug development for patients with squamous NSCLC.

PKA, also known as cAMP-dependent protein kinase A, is not known to be an oncogene. However, growing evidence suggests that this kinase may contribute to cancer progression [6]. For instance, PKA-mediated pathways play an essential role in lung tumorigenesis [7–9]. cAMP response element-binding protein (CREB), a protein downstream of PKA, is a vital transcription factor that controls the expression of several genes, including cell cycle regulators [10].

Functional studies in NSCLC cell lines and genetically engineered lung cancer mouse models of CREB-regulated transcription coactivators support the tumor-promoting role of CREB-mediated transcriptional activation in NSCLC [11–13]. Previous studies have reported that the PKA-CREB axis has tumor-promoting effects in lung cancer [14, 15]. Additionally, inhibiting the PKA-CREB signaling pathway prevents tumor cell proliferation in lung cancer [16].

Several protein kinase inhibitors have been developed to terminate PKA signaling by dephosphorylating PKA substrates [17]. These chemical PKA inhibitors, including cAMP analogs, have been shown to inhibit PKA activity by binding to the ATP-binding pocket of the PKA catalytic subunit [18]. However, small-molecule inhibitors have drawbacks, such as the need for dosage at high concentrations and non-specific off-target effects. Therefore, more research on PKA inhibitors is needed to overcome these drawbacks and improve specificity.

The present study aimed to investigate the efficacy of a novel diarylcyclohexanone derivative, MHY4571, in regulating the PKA-CREB pathway and to study its anti-tumor role in squamous NSCLC. In addition, we also sought to evaluate the synergetic effects of MHY4571 and cisplatin on cell viability in squamous cell lung cancer cell lines.

## Methods

### MHY4571 synthesis

**Fig. 1:**
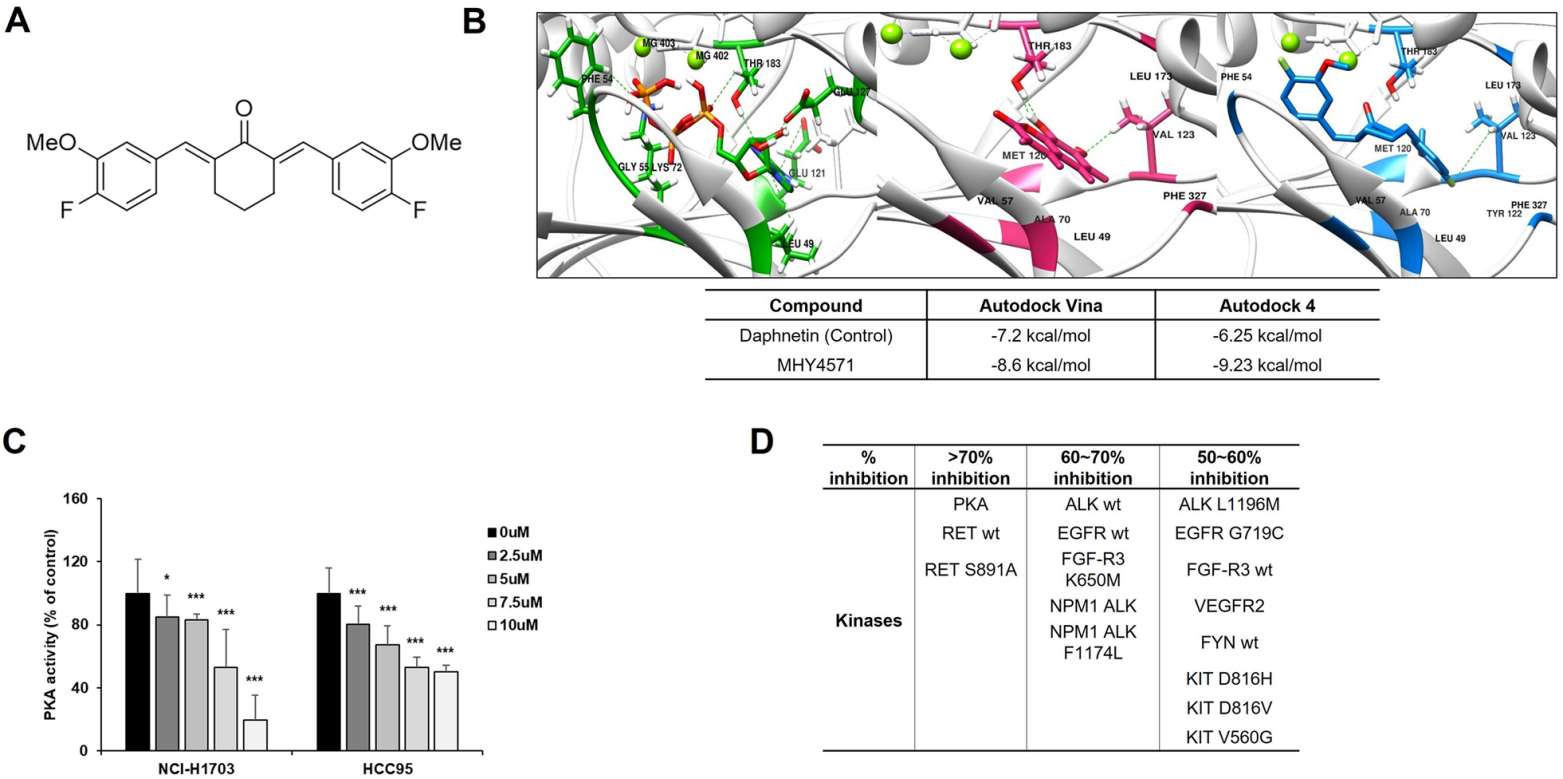
MHY4571 inhibits PKA activity. **A** The chemical structure of MHY4571. **B** Docking simulation of adenosine-triphosphate (ATP) to PKA Cα (PDB code number 4WB5) (left). Simulation of daphnetin to the ATP binding site of PKA Cα (middle). Simulation of MHY4571 to the ATP binding site of PKA Cα (right). Binding affinity to PKA Cα of daphnetin, a known PKA inhibitor and MHY4571 to PKA Cα. **C** PKA kinase activity assay was performed to confirm PKA kinase activity using total cell lysates treated with MHY4571 at 0, 2.5, 5, 7.5, and 10 μM. Data are the means ± SD (*n* = 3). Significance was calculated by the student's t-test (*P < 0.05 and **P < 0.01 *vs*. vehicle-treated cells). **D** Selectivity and target profile of 7.5 μM MHY4571 against 410 kinases in a multikinase inhibition assay in vitro. Shown are kinases whose activity was inhibited by > 50% at the tested dose

A solution of cyclohexanone (0.16 mL, 1.55 mmol) and 4-fluoro-3-methoxybenzaldehyde (471 mg, 3.06 mmol) in a 1 M HCl solution (0.62 mL) was stirred at ambient temperature for 24 h. Cold water was added to the reaction mixture and the resultant solid was filtered and washed with diethyl ether, ethanol, and dichloromethane. The filter cake was further purified by silica gel column chromatography using a mixture of hexane and ethyl acetate (7:1) as an eluent to obtain MHY4571 (411 mg, 73%) as a solid. ^1^H NMR (400 MHz, CDCl_3_) showed peaks at δ 7.68 (s, 2H, 2 × vinylic H), 7.09 – 6.97 (m, 6H, 2′-H, 2′′-H, 5′-H, 5′′-H, 6′-H, 6′′-H), 3.87 (s, 6H, 2 × OCH_3_), 2.87 (t, 4H, J = 5.6 Hz, 3-H_2_, 5-H_2_), 1.77 (quint, 2H, J = 5.6 Hz, 4-H_2_); ^13^C NMR (100 MHz, CDCl_3_) showed peaks at δ 190.0, 152.7 (d, J = 248.8 Hz), 147.7 (d, J = 12.1 Hz), 136.3, 136.0, 132.6 (d, J = 3.7 Hz), 123.4 (d, J = 6.8 Hz), 116.2 (d, J = 18.6 Hz), 116.0, 56.5, 28.5, 23.1; LRMS (ESI +) showed peaks at 393 [M + Na]^+^, 425 [M + MeOH + Na]^+^. The simplified code name and structure of MHY4571 are shown in Fig. 1.

### In silico docking simulation

To determine the possible molecular mechanism of MHY4571 and daphnetin, a known PKA inhibitor, for the inhibition of PKA catalytic subunit alpha, Autodock Vina and Autodock4.2 X-ray crystallographic structures were used to show quaternary ligand binding to aromatic residues in the ATP binding pocket of PKA catalytic subunit alpha (PDB ID: 4WB5) from the RCSB Protein Data Bank (http://www.rcsb.org/adb) [19]. The 3D structure of the two compounds and ATP were created using ChemDraw Ultra. LigandScout 4.1.5 was used to predict possible interactions between MHY4571 and PKA catalytic subunit alpha, and for the identification of pharmacophores [20].

### Chemicals and reagents

All chemicals and reagents were of the purest grade available. Detailed information on the specific antibodies and reagents used in this study is presented in (see Additional file 1: Table S1).

### Cell lines and cell culture

BEAS-2B human lung normal epithelial cells and NCI-H1703 human lung squamous carcinoma cells were obtained from American Type Culture Collection (Manassas, VA, USA). HCC95 human lung squamous carcinoma cells were purchased from the Korean Cell Line Bank (Seoul, Republic of Korea). BEAS-2B cells were cultured using the Bronchial Epithelial Cell Growth Medium Bullet Kit (BEGM; Lonza, Basel, Switzerland) with 10% fetal bovine serum (FBS), 2 mM glutamine, 100 U mL^−1^ penicillin, and 100 μg mL^−1^ streptomycin (WELGENE, Gyeongsan-si, Republic of Korea). Other NSCLC cell lines were grown in RPMI-1640 medium supplemented with 10% FBS, 2 mM glutamine, and penicillin/streptomycin at 37 °C in a humidified atmosphere containing 5% CO_2_. All human cell lines used in this study were authenticated using short tandem repeat profiling (Cosmogenetech, Seoul, Republic of Korea).

### Small interfering RNA transfection

PKA siRNA was purchased from Bioneer (Daedeok-gu, Daejeon, Republic of Korea), and CREB siRNA was purchased from IDT & MBiotech (Coralville, Iowa, USA). Transfection was performed using Lipofectamine RNAiMAX (Invitrogen, MA, USA). Corresponding pairs of scrambled control and siRNA were mixed with Lipofectamine RNAiMAX in Opti-MEM and allowed to react. Cells were treated with a mixture of siRNA and Lipofectamine RNAiMAX in serum-free media and incubated at 37 °C for 48 h.

### MTT assay

Cell viability was determined using the MTT assay. Cells were incubated in the dark with 0.5 mg mL^−1^ MTT at 37 °C for 2 h. Formazan granules generated by live cells were dissolved in DMSO, and the absorbance was measured at 540 nm using a multi-well plate reader (Thermo Fisher Scientific, Waltham, MA, USA). Results are expressed as a percentage of control groups (untreated cells), with untreated cells considered as 100%.

### Annexin V/propidium iodide (PI) double staining

We utilized the BD Pharmingen FITC Annexin V Apoptosis Detection Kit (BD Biosciences, San Diego, CA, USA) to quantitatively determine the percentage of apoptotic cells. Briefly, cells were stained with PI and Annexin V-fluorescein isothiocyanate (FITC) solution at room temperature for 15 min in the dark. The stained cells were then assessed using flow cytometry within 1 h. Flow cytometric analysis was performed using an Accuri C6 flow cytometer (BD Biosciences, Franklin Lakes, NJ, USA).

### Colony-forming assay

Cells were seeded in six-well plates (500 cells well^−1^), incubated overnight, treated with or without cisplatin and/or MHY4571 at the indicated concentrations, and the medium was freshly replaced. Cells were further cultured for ten days and allowed to form clones; the medium was replaced every three days during this time. Subsequently, cells were fixed in 4% paraformaldehyde for 10 min and stained with 0.1% crystal violet for 15 min. Colonies in each treatment group were observed under a microscope and photographed.

### Western blot analysis

Cells were harvested and lysed. Equal amounts of protein were subjected to sodium dodecyl sulfate–polyacrylamide gel electrophoresis and transferred to polyvinylidene fluoride membranes for immunoblotting. The membranes were probed with the relevant primary antibodies overnight, incubated with horseradish peroxidase (HRP)-conjugated secondary antibodies (Enzo Life Sciences, NY, USA), detected using an enhanced chemiluminescence system (Advansta Inc., San Jose, CA, USA), and then monitored using a FUSION Solo-X instrument (VILBER, Wembley, WA, Australia). Densitometric analysis was performed using the ImageJ software (National Institutes of Health, Bethesda, MD, USA).

### Quantitative RT-PCR

RNA was isolated using TRIzol (Sigma, Missouri, USA), and cDNA was synthesized using the cDNA Synthesis Platinum Master Mix (GenDEPOT, Texas, USA). The mRNA was analyzed using the Rotor-Gene Q real-time PCR system. The relative abundance of mRNA was determined after normalization to GAPDH mRNA levels used as a control for experimental variations. The primer sequences used in this study are summarized in Additional file 1: Table S2.

### Kinase profile assay

Kinase selectivity was assessed using the WildType + 2Point-Profiler Assay (ProQinase GmbH, Breisgau, Germany), which included 410 kinases; 13 lipid kinases, as well as disease-relevant kinase mutants, were part of the kinase panel. The assay was performed at a single concentration (7.5 µM) using ATP K_m_ for each kinase.

### PKA kinase activity

PKA activity was evaluated using a PKA Kinase Activity Kit (Enzo Life Sciences, NY, USA). This assay is based on a solid-phase enzyme-linked immunosorbent assay (ELISA) that utilizes a specific synthetic peptide as a substrate for PKA and a polyclonal antibody that recognizes the phosphorylated form of the substrate. Samples (cell lysates) and diluted standard active PKA were added to the PKA substrate microtiter plate. After adding ATP to each well containing the sample, the plate was incubated at 30 °C for 90 min. Each well was emptied, and the phosphor-specific substrate antibody was added and incubated for 60 min at room temperature. After washing each well four times, peroxidase-conjugated secondary antibodies were added and incubated for 30 min at room temperature. Subsequently, wells were washed four times, and tetramethylbenzidine substrate was added. Color development was stopped with an acid stop solution. The color (which is proportional to the PKA phosphotransferase activity) was measured in a microplate reader at 450 nm.

### Xenograft mice model

All animal protocols conformed to guidelines for the care and maintenance of laboratory animals [21]. Female BALB/c nude mice were obtained from OrientBio (Kyunggi, Republic of Korea) and used for experiments after they were 5–6 weeks old. Animals were acclimated for seven days and weighed at the end of the acclimation period. NCI-H1703 cells were subcultured, stained with trypan blue, and counted to assess cell viability. These were then transplanted into the right dorsal flanks of mice (5 × 10^6^ cells/animal; n = 24). Mice were assigned to each of the following groups: vehicle (n = 6) and three different MHY4571 concentration groups (5 mg kg^−1^ vs. 10 mg kg^−1^ vs. 20 mg kg^−1^) (n = 6 per group). The oral route of drug administration was used, as this administration route is used in clinical practice. MHY4571 was administered orally five times per week for three weeks. The mean body weight was measured for each group after the group assignment. Tumor volumes were measured with digital calipers and calculated according to the formula: 0.52 × length × width^2^, and tumor masses were evaluated at 3-weeks post-injection. Mice were sacrificed at the end of the study by being placed in a CO_2_ chamber.

### Orthotopic mouse model

Male BALB/c nude mice were obtained from ORIENT BIO, Inc. (Seongnam-si, Gyeonggi-do, Republic of Korea). The constructed NCI-H1703/f-Luc-GFP cells (Lugen Sci Co, Gyeonggi-do, South Korea) were subcultured, stained with trypan blue, and counted to assess cell viability. These cells were then suspended in 30 μL HBSS and loaded into a 29G syringe for lung administration at 4 × 10^7^ cells/ml. These mice were monitored to visualize the growth of the tumor cells using an IVIS imaging system on weekly basis (IVIS Spectrum CT, Perkin Elmer). 100 uL of D-luciferin (30 mg/ml) was injected intra-peritoneally in mice 12 min before bioluminescence imaging. Mice were assigned to each of the following groups: vehicle (n = 5), low-dose MHY4571 (10 mg kg^−1^) (n = 5), and high-dose MHY4571 (20 mg kg^−1^) (n = 5). The mean body weight was measured for each group after the group assignment. Mice were divided into groups of five per cage. All animals were euthanized with CO_2_ asphyxiation after the termination of the experiment. For the observation of lung cancer, the lungs were fixed with Bouin’s solution.

### Tissue microarray construction

Tumor tissue microarray was constructed using two tissue cores (3-mm diameter) per tumor to obtain tissue from central and peripheral tumor areas after histologic examination of 236 NSCLC specimens in the Pathology Department of Pusan National University Yangsan Hospital. All tumors were histologically examined and classified using the 2015 World Health Organization International Classification of Lung Tumors.

### Immunohistochemistry (IHC)

For immunohistochemical analysis, consecutive 3 µm-thick tissue sections were cut from formalin-fixed paraffin-embedded tissues. IHC staining was performed with the primary antibody (p-PKA, 1:200; p-CREB, 1:200) overnight at 4 °C. Subsequently, 3,3'-diaminobenzidine staining and TUNEL assays were performed using an ApopTag Fluorescein Direct In Situ Apoptosis Detection Kit (Millipore, Billerica, MA, USA). Hematoxylin and eosin- and IHC-stained slides were digitally scanned with a Zeiss Axio Scan Z1 microscope using ZEN 2.3 software (Carl Zeiss Microscopy GmbH, Jena, Germany).

### Kaplan–Meier plotter

The Kaplan–Meier Plotter was used to assess the prognostic significance of PKA catalytic subunit alpha in squamous cell lung carcinoma. First, datasets (GSE17710, n = 56; GSE4573, n = 130) for prognostic analysis were obtained from Gene Expression Omnibus (GEO) of the National Center for Biotechnology Information (https://ncbi.nlm.nih.gov/geo). Based on PRKACA expression, patients were divided into three groups (low, medium, and high). Using Cox proportional hazard models, KM plots for overall survival and relapse-free survival of the patients with lung squamous cell carcinoma were conducted. KM curve and statistical analysis were performed using SPSS programs (version 20).

### Combination index (CI) analysis

The CI method proposed by Chou-Talalay, based on the median-effect equation of the mass-action law, allows for the quantitative computerized simulation of synergy (CI < 1), additive effect (CI = 1), and antagonism (CI > 1) at all dose and effect levels in vitro or in vivo for various types of drugs, including radiation [22]. This analysis was applied using CompuSyn software, 2005 (www.combosyn.com).

### Statistical analysis

Statistical analysis for in vivo experiments was performed using Prism 8 software (GraphPad Software Inc., CA, USA). For comparisons of more than two groups, one-way ANOVA was performed, followed by a posthoc test with Dunnett’s correction of pairwise group differences. All data are expressed as the mean ± standard deviation (SD) of three experiments and were analyzed using the student’s t-test. Means were considered significantly different at *P < 0.05, **P < 0.01, and ***P < 0.001.

## Results

### Development of MHY4571 and evaluating its kinase inhibition profile

To identify novel PKA inhibitors, we initially designed several molecules based on the well-known PKA inhibitor H89 structure and then screened the molecules using a computerized docking simulation. This investigation led to the identification of four compounds, among which a novel diarylcyclohexanone derivative (MHY4571; Fig. 1A) was identified as a PKA inhibitor with favorable in silico absorption, distribution, metabolism, and excretion (ADME) properties (see Additional file 1: Table S3). To investigate the mechanism whereby MHY4571 inhibited PKA activity, we conducted two different computerized docking simulations. Daphnetin, a known PKA inhibitor [23], preferentially binds to the ATP-binding site in the PKA catalytic alpha subunit structure (Fig. 1B). Docking simulation of MHY4571 to the ATP binding site revealed that MHY4571 binds to the same site as daphnetin. The relative docking affinities of MHY4571 (-8.6 kcal mol^−1^ for Autodock Vina and -9.23 kcal mol^−1^ for Autodock 4) to the ATP binding site were close to those of daphnetin (-7.2 kcal mol^−1^ for Autodock Vina and -6.25 kcal mol^−1^ for Autodock 4). To verify the PKA kinase inhibitory activity of MHY4571, we assessed the PKA kinase activity. Results from the assays showed that PKA activity was reduced in the lung cancer lines NCI-H1703 and HCC95 upon treatment with MHY4571 (Fig. 1C). In addition, MHY4571 also inhibited other kinases, as assessed by a multikinase inhibition assay, including a panel of 410 kinases (Fig. 1D and see Additional file 2: Table S4). The inhibitory activities of MHY4571 against wild-type RET and RET S891A were comparable to that against PKA in an assay that used 7.5 μmol L^−1^ of ATP.

### Overexpression of PKA activity in squamous NSCLC

**Fig. 2:**
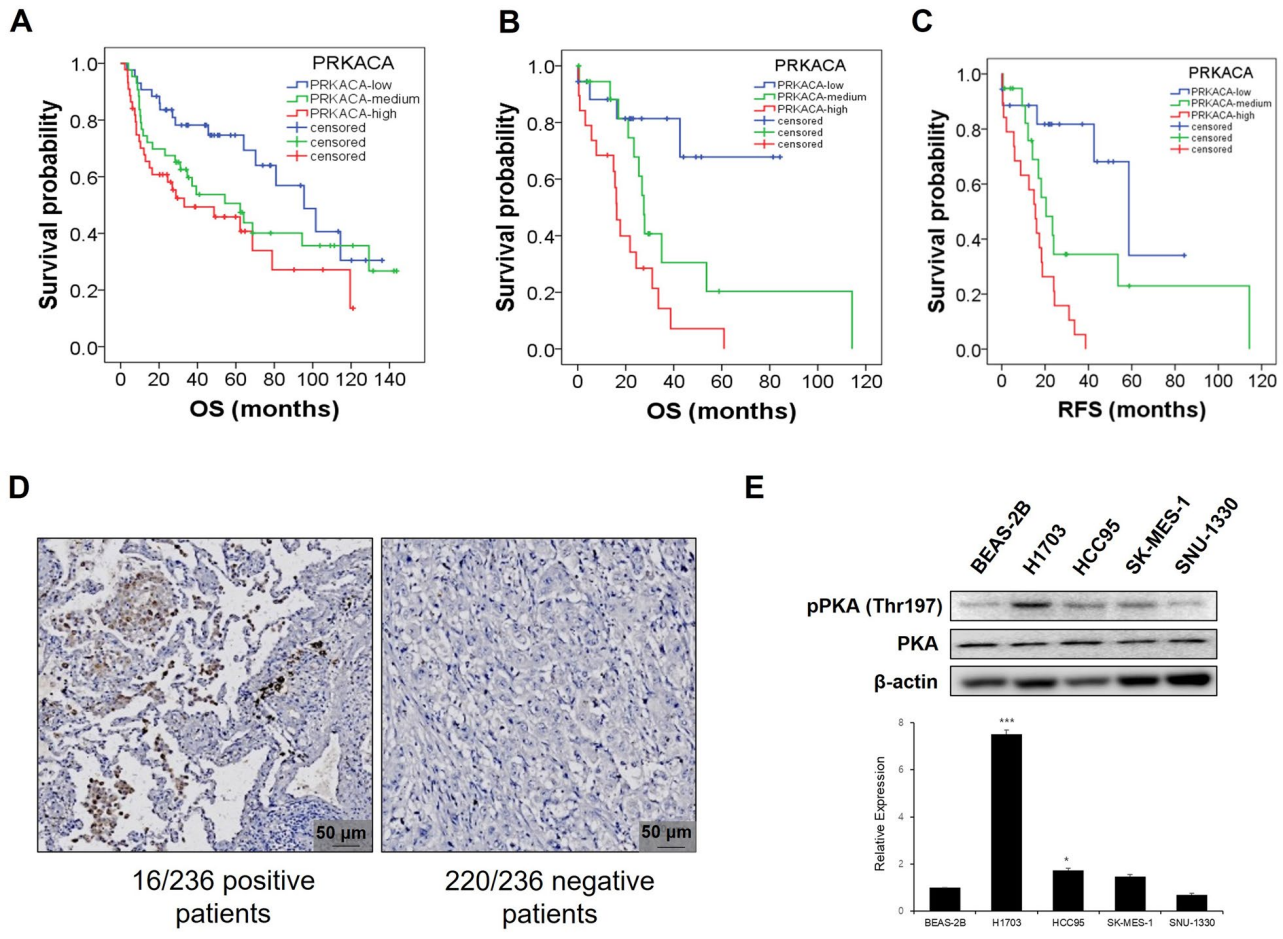
Overexpression of PKA catalytic subunit alpha and its clinical significance Kaplan–Meier analysis of overall survival and relapse-free survival by low, medium, and high PKA Cα (PRKACA) expression. Overall survival analysis (**A**, GSE4373, p = 0.024; **B**, GSE17710, p = 0.001) and relapse-free survival (**C**, GSE17710, p < 0.001) of the patients with squamous NSCLC were performed using Cox proportional hazard models and follow-up data for the indicated period. The result was as follows. The blue line indicates the group with low PRKACA expression ((a) n = 43, (b) n = 18, (c) n = 18) and the green line indicates the group with medium PRKACA expression ((a) n = 43, (b) n = 19, (c) n = 19). The red line indicates the group with low PRKACA expression ((a) n = 44, (b) n = 19, (c) n = 19). **D** Representative immunohistochemical analysis for PKA activity scored by P-PKA Cα substrate in the tissue microarray constructed using samples obtained from 236 NSCLC patients. Scale bar = 50 μm. **E** Total cell lysates of normal human lung epithelial BEAS-2B cells and four different squamous-cell lung carcinoma (SQC) cell lines (NCI-H1703, HCC95, SK-MES-1, and SNU-1330) were subjected to western blot analysis for the expression levels of P-PKA and PKA Cα. Results are expressed as the means ± SD of three individual experiments. Significance was calculated by the student's t-test (*P < 0.05, **P < 0.01, and ***P < 0.001, *vs*. lung normal epithelial cells). Data are the means ± SD (n = 3)

Previously, the PKA catalytic subunit alpha (PRKACA) was identified as the dominant catalytic subunit that is expressed and active in lung cancer; it is reported to be essential for tumor development and growth [24]. To determine the survival benefits of PKA catalytic subunit alpha in patients with squamous cell lung carcinoma, Kaplan–Meier analysis of OS and RFS was performed. Both OS (Fig. 2A/B) and median RFS (Fig. 2C) showed significant clinical benefits according to the dataset of patients with squamous cell lung carcinoma obtained from GEO. We analyzed NSCLC human tissue microarrays by immunostaining with the P-PKA antibody and found that 6.7% of the human tumors examined showed positive staining for P-PKA (Fig. 2D). In analyzing the expression levels of PKA catalytic subunit in human squamous lung cancer cell lines, western blot showed a consistent PKA level and revealed that the phosphorylation level of PKA (p-PKA) was higher in squamous cell lung carcinoma cells (NCI-H1703 and HCC95) than in a normal broncho-epithelial cell line (BEAS-2B) (Fig. 2E).

### Treatment with MHY4571 inhibits the proliferation of squamous cell lung cancer cells

**Fig. 3:**
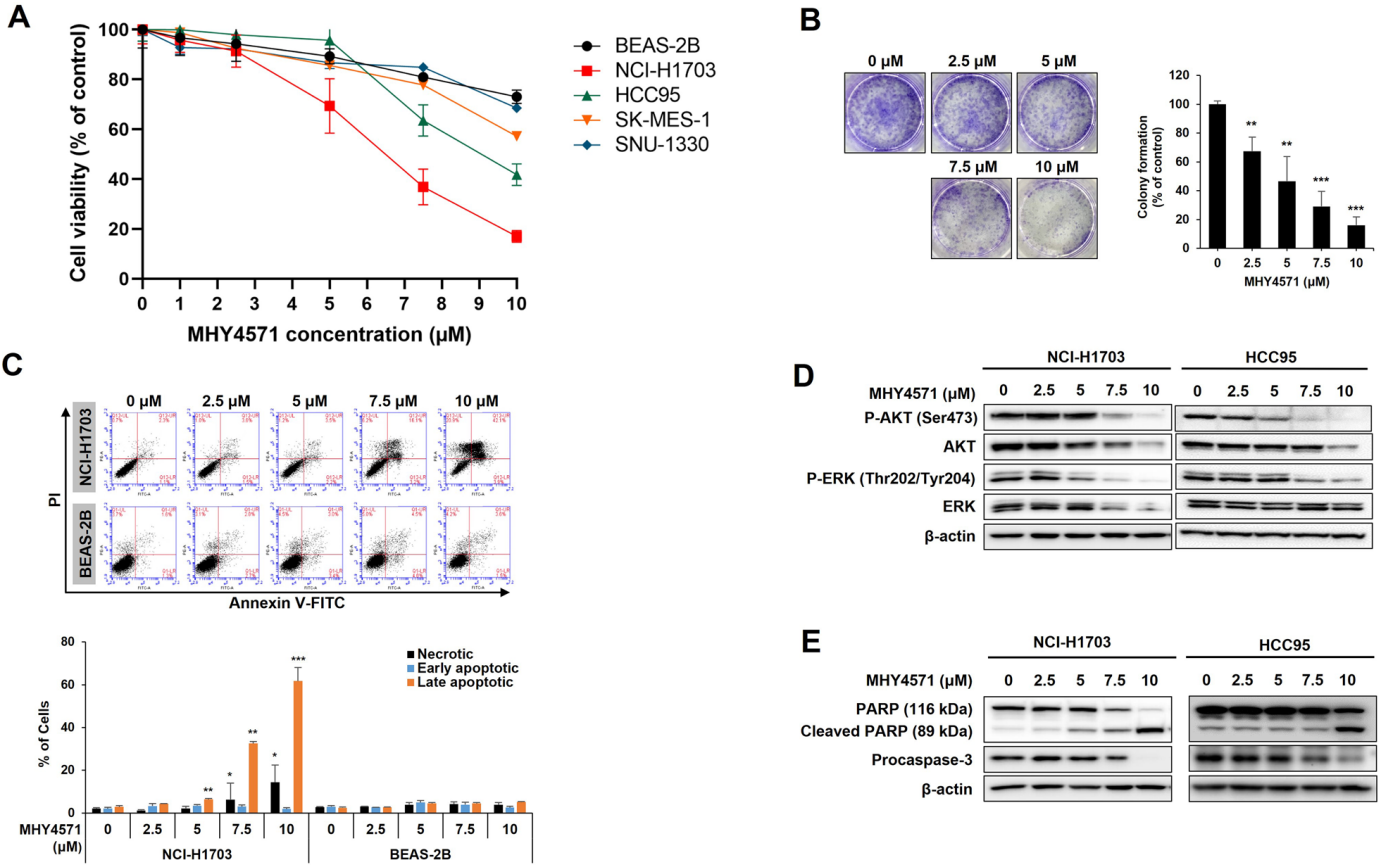
MHY4571 induces apoptosis in human lung cancer cells (**A**). The normal broncho-epithelial cells (BEAS-2B) and four different human squamous lung cancer cells were treated with increasing concentrations of MHY4571 for 24 h, and then the percentage of cell viability was determined by the MTT assay. **B** NCI-H1703 cells were treated with MHY4571 for 8 h, and the medium was replaced with a fresh medium. After incubation for 10 days, colonies were stained with crystal violet. **C** NCI-H1703 cells were treated with variable concentrations of MHY4571 for 24 h. The cells were stained with Annexin V-FITC/PI and analyzed by flow cytometry. **D** NCI-H1703 cells and HCC95 were incubated with MHY4571 as indicated for 24 h. The blots were probed with antibodies against P-Akt, Akt, P-ERK, and ERK. β-actin was used as an internal control. **E** PARP and procaspase-3 were detected to determine the apoptotic effects of MHY4571. Data are the means ± SD (n = 3). Significance was calculated by the student's t-test (*P < 0.05, **P < 0.01, and ***P < 0.001 *vs*. vehicle-treated cells)

As the PKA-mediated signaling pathway was reported to have a tumor-promoting effect [8], we hypothesized that elevated PKA activity would be associated with squamous NSCLC cell proliferation and that the regulation of PKA activity by MHY4571 would inhibit cancer growth. Accordingly, to assess the anti-proliferative activity of MHY4571, its growth-inhibitory effects were investigated in squamous lung cancer cell lines and a normal bronchial epithelial cell line using the MTT assay. The lung cancer cell lines showed varied responses to MHY4571; however, the response was minimal in BEAS-2B cells (Fig. 3A). The NCI-H1703 cell line was chosen for further experimentation as it was sensitive to treatment, and BEAS-2B was selected as the control cell line. MHY4571 showed anti-proliferative effects in the colony formation assay (Fig. 3B) and the Annexin V apoptosis assay (Fig. 3C). MHY4571 inhibited the phosphorylation of AKT and ERK (Fig. 3D). MHY4571 treatment induced caspase-3 and PARP cleavage and enhanced cell death (Fig. 3E).

### MHY4571 reduces cell viability by repressing PKA-CREB signaling

**Fig. 4:**
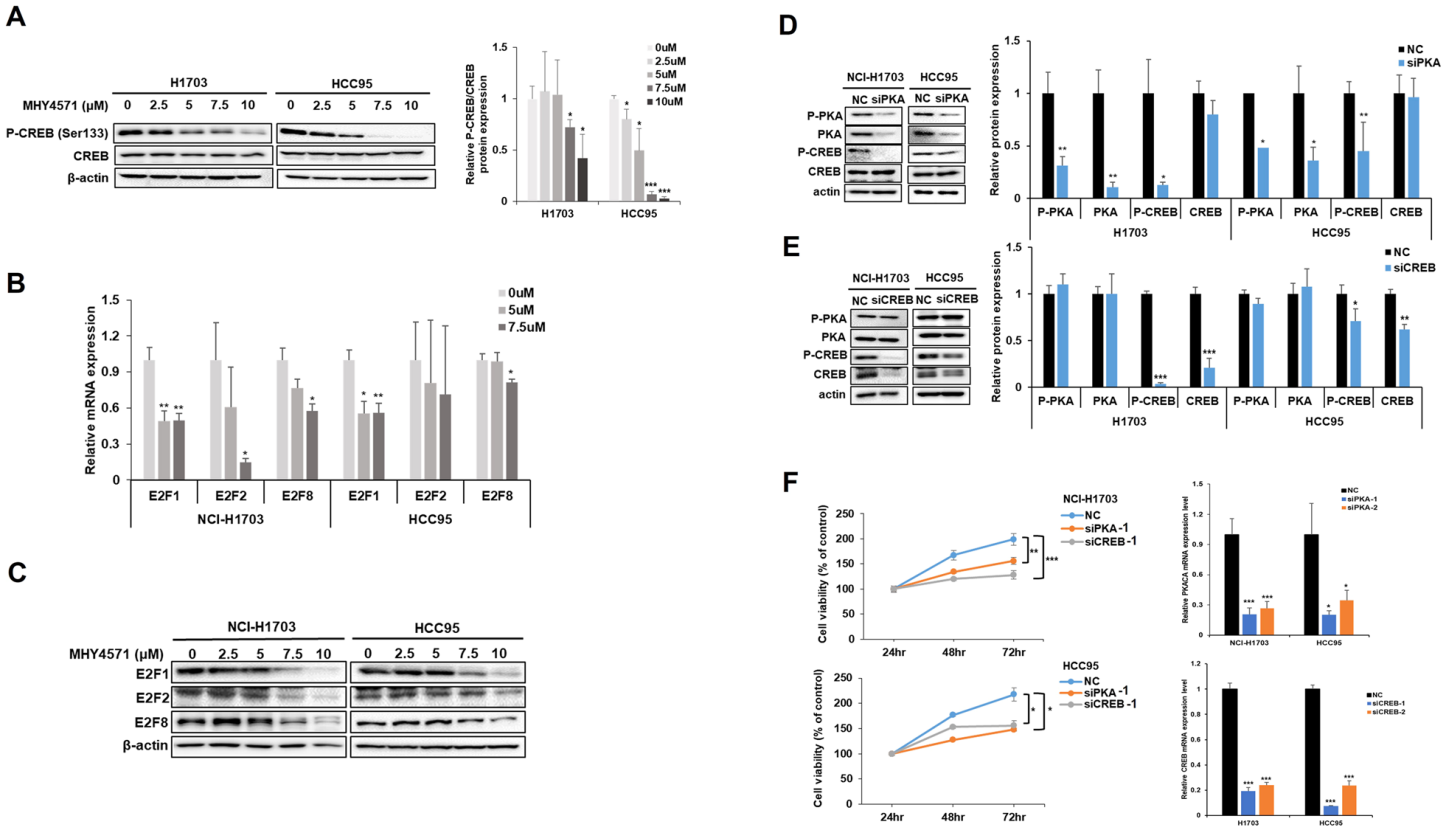
MHY4571 induces apoptosis through, at least in part, the PKA/CREB axis in human squamous carcinoma lung cancer cells. **A** NCI-H1703 cells and HCC95 cells were treated with indicated concentrations of MHY4571 for 24 h, and western blot analyses were conducted to investigate the expression of P-CREB and CREB. **B**, **C** NCI-H1703 cells and HCC95 cells were treated with MHY4571 at 0, 5, and 7.5 µM for 24 h, and then the mRNA expression levels of E2F1, E2F2, and E2F8 were analyzed by quantitative RT-PCR and western blot analyses. β-actin was used as an internal control. Significance was determined using student's t-test (*P < 0.05, **P < 0.01, and ***P < 0.001 *vs*. vehicle-treated cells). **D**, **E** NCI-H1703 cells and HCC95 cells were transfected with siPKA, siCREB, and scramble siRNA (negative control) for 48 h, and then the expression levels of P-PKA, PKA, P-CREB, and CREB proteins were analyzed by western blot. β-actin was used as a loading control. Significance was determined using student’s t-test (*P < 0.05 and ***P < 0.001 *vs*. scramble siRNA transfected cells). **F** NCI-H1703 cells and HCC95 cells were transfected with siPKA, siCREB, and scramble siRNA (NC) for 24, 48, and 72 h, and then MTT assay was performed to confirm cell viability. Results are expressed as the means ± SD of three individual experiments. Significance was determined using student’s t-test test (*P < 0.05, **P < 0.01, and ***P < 0.001 *vs*. scramble siRNA transfected cells (NC))

To examine the association between PKA signaling and lung cancer cell proliferation, we used two squamous NSCLC cell lines, NCI-H1703, and HCC95. We first confirmed that treatment with MHY4571 reduced the p-CREB-to-CREB ratio in a dose-dependent manner (Fig. 4A; Additional file 1: Fig. S1). We also determined the active transcription of CREB target genes, E2F family members, E2F1, E2F2, and E2F8. We found that E2F mRNA and proteins were decreased by the treatment with MHY4571 in dose-dependent manner (Fig. 4B & C).

We then evaluated whether PKA was an upstream regulator of CREB and examined the functional consequences of inactivating PKA or CREB to better understand the function of these pathways in promoting lung cancer cell proliferation. We performed western blot assay in cancer cells transfected with siPKA or siCREB (transfected cells vs. scrambled control cells (siControl)). We found that siPKA-treated cells showed decreased levels of p-PKA and p-CREB, but siCREB-treated cells showed a downregulation of p-CREB alone, suggesting that PKA is an upstream modulator of CREB (Fig. 4D & E). Next, we examined whether PKA-CREB signaling is associated with cell viability. We found that siPKA- or siCREB-transfected cells showed decreased viability compared to scrambled control-treated cells (Fig. 4F).

### MHY4571 significantly suppresses tumor growth

**Fig. 5:**
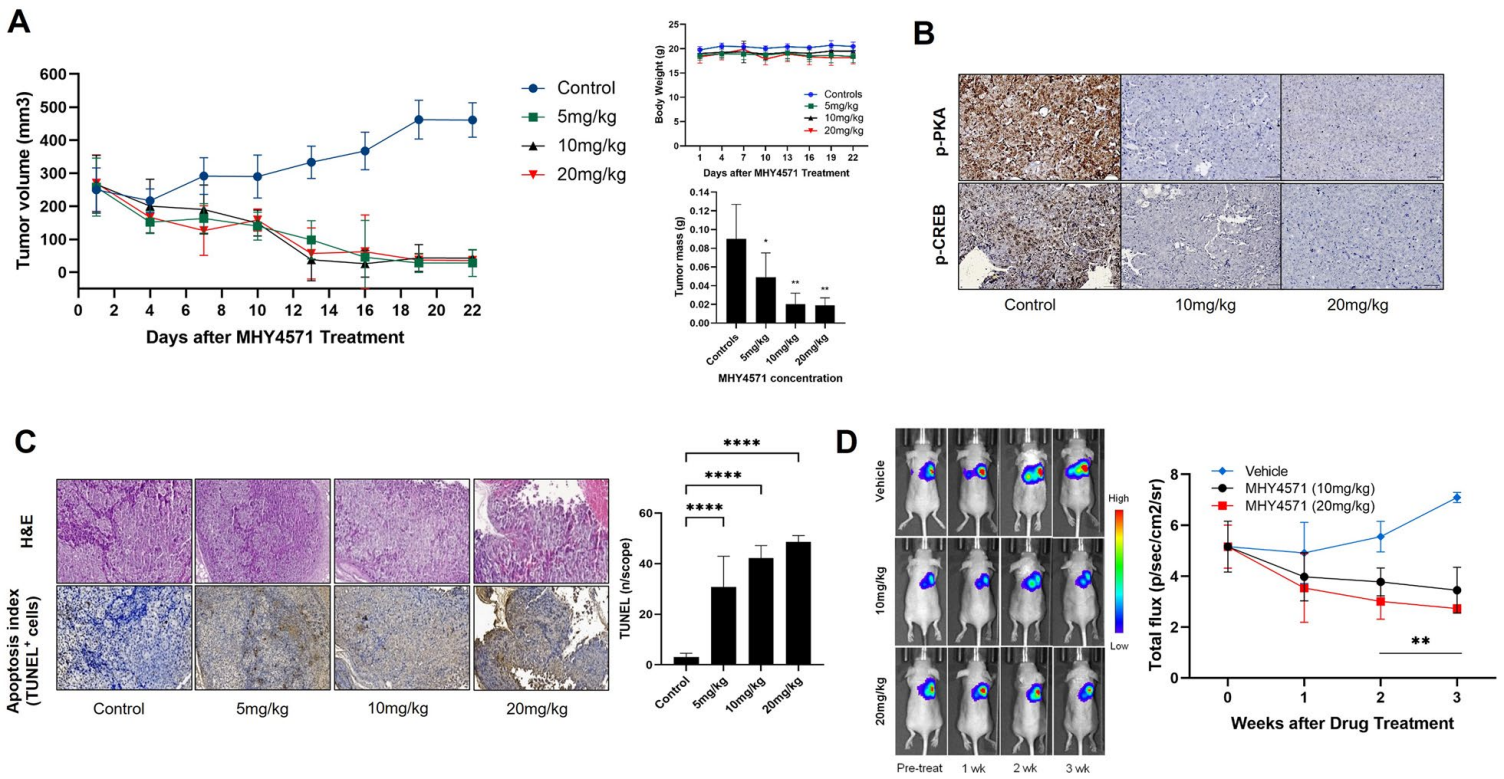
MHY4571 inhibits tumor progression in a mouse xenograft and orthotic model. **A** After NCI-H1703 cell injection, mice harboring human lung tumors were treated with vehicle (control) or MHY (5, 10, and 20 mg kg^−1^). Tumor growth was measured in control-treated and MHY-treated groups (n = 6 control; n = 6 for each MHY concentration). Body weight and tumor mass were measured. **B** Immunohistochemical staining for p-PKA and p-CREB, as described in the Methods. **C** H&E staining and TUNEL assays were performed in control-treated and MHY-treated groups. Data are shown as the means ± SD. Statistical significance is indicated as **** P < 0.0001. **D** Representation of the bioluminescent signal at pre-treatment, 1 week, 2 weeks, and 3 weeks after MHY treatment (10 mg kg^−1^; n = 5 and 20 mg kg^−1^; n = 5). Statistical significance is indicated as ** P < 0.01

To determine whether MHY4571 inhibits in vivo tumor growth, we used NCI-H1703 xenograft and orthotopic mouse models. The tumor volumes and masses in mice administered MHY4571 (5 mg kg^−1^, 10 mg kg^−1^, and 20 mg kg^−1^ daily) were significantly smaller than those in the vehicle control group (Fig. 5A). Additionally, MHY4571 was well tolerated at these doses (Fig. 5A). Immunohistochemical analysis for p-PKA and p-CREB (a well-known target of PKA) showed that control tumors (untreated vehicle controls) showed subpopulations of p-PKA- and p-CREB-positive cells; however, treatment with MHY4571 reduced p-PKA and p-CREB positivity (Fig. 5B). The results of the TUNEL assay showed that MHY4571 treatment led to an increase in the number of apoptotic cells (Fig. 5C).

Because the two high doses (10 mg kg^−1^ and 20 mg kg^−1^) did not show any unacceptable side effects or toxicities in experimental xenograft mice, we further examined the effects of MHY4571 at these two doses on the growth of orthotopically implanted lung cancer tumors. The bioluminescence imaging results indicated a gradual increase in tumor volume in the control group compared with that in the two treatment groups. The final tumor volumes on day 21 after the start of treatment were lower than those in the control group (P < 0.05, treatment vs. control; Fig. 5D). There was a corresponding result for tumor volume treated with 10 mg kg^−1^ and 20 mg kg^−1^ MHY4571.

### Effect of combined treatment with cisplatin and MHY4571 in squamous cell lung cancer cells

**Fig. 6:**
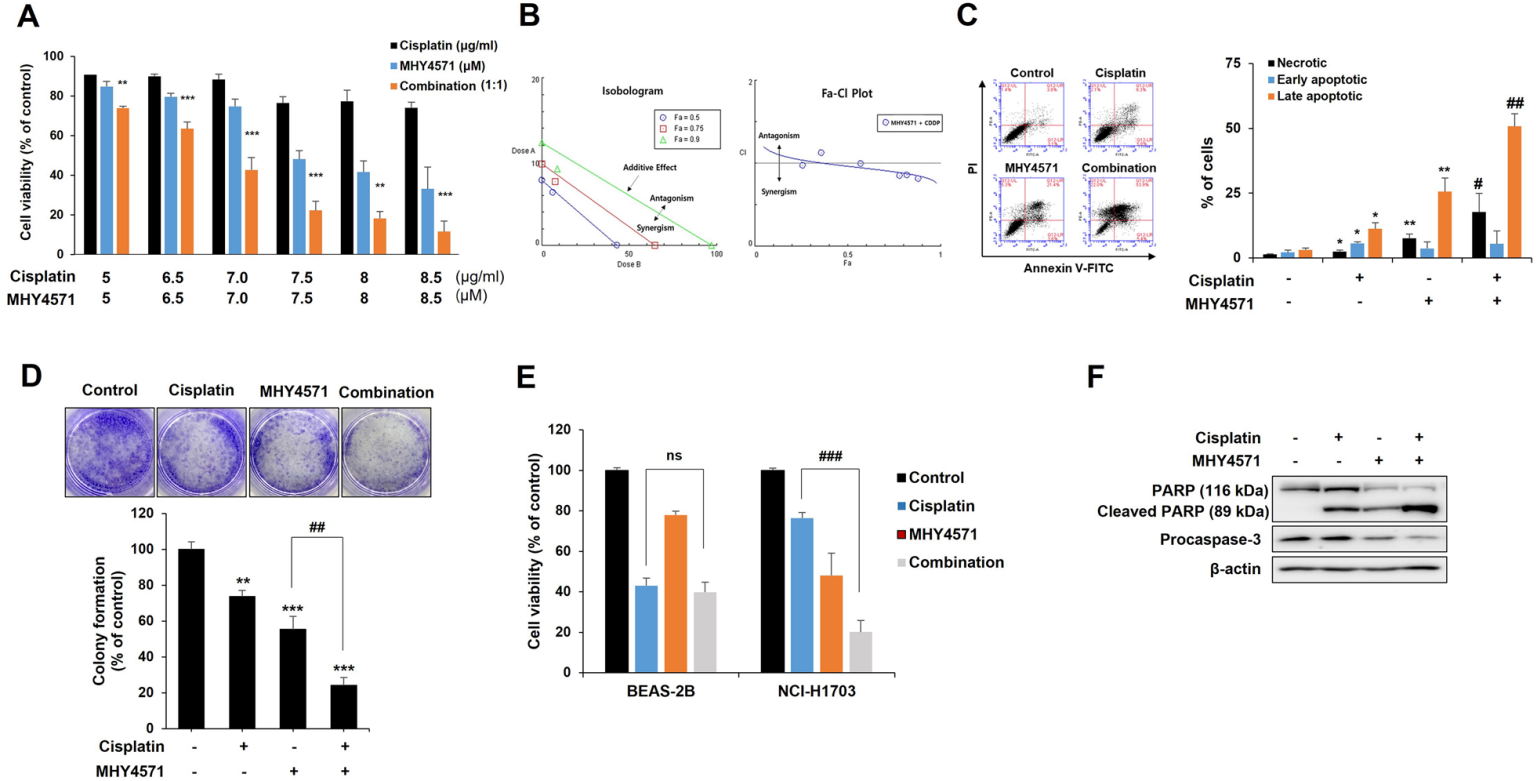
Effect of combined treatment with cisplatin and MHY4571 in NCI-H1703 cells. NCI-H1703 cells were incubated for 24 h treatment with 1:1 drug mixture (cisplatin/MHY4571) in increasing concentrations. Combined effects were determined using **A** MTT assay and **B** CI values. Results are mean ± SD, n = 3, expressed as a percentage of that in vehicle-treated control cells. (*P < 0.05, **P < 0.01, and ***P < 0.001 *vs*. MHY4571-treated cells). **C** Annexin V-FITC binding and PI uptake in non-permeabilized cells were analyzed by flow cytometry. **D** Colony formation assays of crystal violet-stained colonies. *P < 0.05, **P < 0.01, and ***P < 0.001 vs. vehicle-treated cells; #P < 0.05 and ##P < 0.01 vs. MHY4571-treated cells (student’s t-test). **E** NCI-H1703 cells and BEAS-2B cells were incubated with or without 7.5 μg/mL cisplatin and/or 7.5 μM MHY4571 for 24 h, and combined effects were assessed using MTT assay. *###*P < 0.001 for the combination of cisplatin plus MHY4571 *vs*. cisplatin-treated cells (student’s t-test). **F** Total proteins were prepared and immunoblotted for PARP, procaspase-3, P-Akt, Akt, P-ERK, and ERK. β-actin was used as a loading control

Next, we investigated whether combined treatment with MHY4571 and cisplatin had synergistic effects on cell death. We used compusys, a publicly available database, to determine the synergistic effects and found that co-treatment synergistically increased apoptosis based on the results of the MTT assay (Fig. 6A and B). Similar synergistic effects were observed in the Annexin V-FITC apoptosis assay (Fig. 6C) and colony formation assay (Fig. 6D). We then evaluated whether the combination of MHY4571 and cisplatin induced additional cytotoxicity in the normal epithelial cell line BEAS-2B. Our results showed that the co-treatment did not significantly increase cell death in BEAS-2B cells (Fig. 6E). Furthermore, the combination treatment induced caspase-3 and PARP-1 cleavage and inhibited the phosphorylation of AKT and ERK (Fig. 6F). All the above effects showed dose-dependent trends. These findings demonstrate that MHY4571 has dose-dependent and caspase-3-dependent cytotoxic effects in lung cancer cells but not in normal epithelial cells.

## Discussion

In this study, we designed and screened, in silico, several novel PKA inhibitors and identified MHY4571 as a potentially viable PKA inhibitor with favorable in silico ADME properties. Our data from in vitro and in vivo experiments revealed that MHY4571 functions as a novel PKA inhibitor in NSCLC and exerts anticancer effects mediated by the inactivation of the PKA-CREB signaling pathway. Our findings indicate that PKA could be a therapeutic target for squamous cell lung carcinoma, and the suppression of PKA activity by MHY4571 could potentially be employed to treat this cancer type.

The “druggable” driver oncogenes in NSCLC that have been successfully targeted include epidermal growth factor receptor (EGFR), anaplastic lymphoma kinase, BRAF, RET, and ROS1. Newly developed antibody–drug conjugates and small molecule inhibitors of other oncogenes, such as cMET and Kirsten rat sarcoma virus (KRAS), are currently being tested in clinical trials. The incidence of these driver oncogenes is approximately 1–10%, except for EGFR and KRAS, which have relatively high incidences of up to 20–30% [25]. In this study, we used tissue microarrays to evaluate the expression of p-PKA in NSCLC cells. Our data revealed that 16 of 236 NSCLC specimens (6.7%) were p-PKA-positive; this number was comparable to that for the other driver oncogenes. Our data and previous studies [10, 24] indicate that a high level of PKA activity is associated with a poor prognosis and OS in lung cancer. This suggests that PKA activity, at least in part, is required for cell proliferation in NSCLC.

Using the structure of the well-known PKA inhibitor H89 as the starting point, we designed novel PKA inhibitors. As shown in Additional file 1: Fig. S2, H89 consists of two aromatic rings (A and C) connected by a linker (B). Based on the structural characteristics of H89, several diaryl derivatives with 2,6-dimethylenecyclohexanone or 2,5-dimethylenecyclopentanone moieties as linkers were designed and synthesized. Based on the preliminary screening results, (2E,6E)-2,6-bis(4-fluoro-3-methoxybenzylidene) cyclohexanone (MHY4571) was selected for further experiments. We also compared the effectiveness of MHY4571 to H89 and found that MHY4571 has relatively more cytotoxic effects in PKA overexpressed H1703 cells (Additional file 1: Fig. S3). Results from the docking simulation analysis suggest that MHY4571 binds to the ATP pocket of the PKA catalytic subunit. We then performed protein kinase assays to investigate whether MHY4571 exerts an inhibitory effect on the kinase activity by reducing ATP concentrations. Results from protein kinase assays involving the measurement of radioactive ATP concentrations showed that MHY4571 reduced PKA activity. Western blot analysis showed that MHY4571 tends to inhibit PKA phosphorylation (Additional file 1: Fig. S4). However, further studies are warranted to confirm the phospho-PKA level and its particular phosphorylated site. Kinase inhibition assay results showed that MHY4571 also inhibited other kinases to a lower degree (< 70%); the exception was RET (wild-type and S891A), which showed a > 70% inhibition (Fig. 1D). However, MHY4571 did not improve cell viability in the LC-2/ad cell line harboring a *CCDC6-RET* fusion; this genetic alteration is the most frequent RET rearrangement observed in NSCLC [26] (Additional file 1 Fig. S5). In addition to selectivity concerning kinases, it is also essential to focus on selectivity for tumor cells compared to normal cells. Our data showed that MHY4571 had minimal effect on the viability of normal cells (BEAS-2B), while it was highly effective in reducing the viability of lung cancer cell lines.

Our data demonstrated that the downregulation of p-PKA by MHY4571 suppressed NSCLC tumorigenesis, suggesting a tumor-suppressive function for PKA in NSCLC cell lines, and in vivo tumor mass. These findings are supported by previous studies that reported that the inhibition of PKA leads to antitumor effects in lung cancer [14]. We also found that combination treatment with MHY4571 and cisplatin synergistically reduced cell viability. Further experimentation is warranted to determine the optimal dose of MHY4571 and cisplatin that is efficacious and does not show adverse effects when used as part of the combination therapy.

Interestingly, it has been observed that activating PKA can induce tumor cell proliferation through CREB activation in lung cancer [27]. MHY4571 exerted tumor-suppressive functions through the PKA-CREB signaling pathway in vitro and NSCLC tumors in vivo. Previous studies have revealed that the activation of PKA, the mitogen-activated kinases ERK1/2, and CREB is associated with the proliferation of human lung adenocarcinoma cells [14]. Downregulation of the PKA-CREB pathway inhibited hypoxia-induced epithelial-mesenchymal transformation, cell migration, and invasion of lung cancer cells [28]. These results suggest that the PKA-CREB pathway plays an essential role in the proliferation of cancer cells, and this pathway can be targeted for the treatment of lung cancer. E2F1, E2F2, and E2F8 are E2F family members and are overexpressed in lung cancer cells (compared to normal human lung tracheobronchial epithelial cells). These E2Fs play a critical role as target genes of CREB in the growth of lung cancer cells [29]. This led us to hypothesize that the MHY4571-mediated downregulation of the PKA-CREB pathway may be associated with cell cycle arrest via modulating molecules such as E2Fs. Consistent with a previous study, we found that the overexpression of E2Fs was abrogated by MHY4571 treatment. Future studies should focus on elucidating the detailed mechanisms underlying the PKA-CREB-mediated regulation of E2Fs and the eventual suppression of NSCLC cell viability.

The limitation of this study was that we used the siRNA technique for gene-silencing of PKA catalytic subunit alpha. The siRNA method has variable effects in terms of the extent of gene knockdown and could generate off-target effects through a different mechanism. Thus, for future detailed mechanistic studies, gene knockout techniques, such as the CRISPR/cas9 method, are warranted to knock out PKA expression.

## Conclusions

MHY4571, a novel diarylcyclohexanone derivative, exerts antitumor effects in NSCLC by inhibiting the PKA-CREB pathway. Therefore, MHY4571 may be a potential therapeutic agent for patients with NSCLC.

## Supplementary Information

**Additional file 1: Figure S1.** Design of a novel PKA inhibitor, MHY4571, from the structure of the well-known PKA inhibitor H89. **Figure S2**. Effect of MHY4571 on cell viability of LC-2/ad cells. **Table S1**. Key resources table. **Table S2.** Primer sequence. **Table S3**. In silico ADME for MHY4571.

**Additional file 2: Table S4.** The kinase inhibition profile of MHY4571.

## Data Availability

The datasets used and/or analysed during the current study are available from the corresponding author on reasonable request.

## Abbreviations

NSCLC: Non-small cell lung cancer
CREB: CAMP response element-binding protein
ADME: Absorption: distribution: metabolism: and excretion
PRKACA: PKA catalytic subunit alpha
EGFR: Epidermal growth factor receptor
KRAS: Kirsten rat sarcoma virus
IHC: Immunohistochemistry
PI: Propidium iodide
