# Supplementary material for: MHY4571, a novel diarylcyclohexanone derivative, exerts anti-cancer activity by regulating the PKA-cAMP-response element-binding protein pathway in squamous cell lung cancer
Source: Exp Hematol Oncol. 2022 Oct 8;11:68. doi: 10.1186/s40164-022-00324-8 (PMC9547450; doi:10.1186/s40164-022-00324-8)
Supplement: Supplementary file 1 — Additional file 1: Figure S1. Design of a novel PKA inhibitor, MHY4571, from the structure of the well-known PKA inhibitor H89. Figure S2. Effect of MHY4571 on cell viability of LC-2/ad cells. Table S1. Key resources table. Table S2. Primer sequence. Table S3. In silico ADME for MHY4571. [file 40164_2022_324_MOESM1_ESM.docx]

**Supplementary material**

**
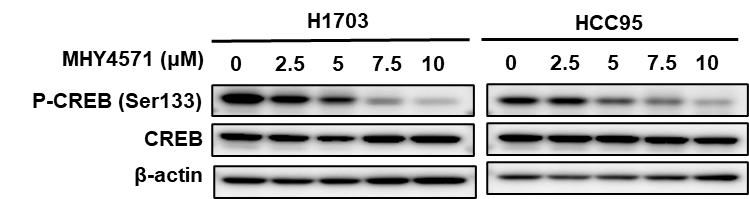
**

**Supplementary Fig. 1. Western blot analysis to determine CREB and p-CREB levels after treatment of MHY4571.** NCI-H1703 cells and HCC95 cells were treated with indicated concentrations of MHY4571 for 24 h, and western blot analyses were conducted to investigate the expression of P-CREB and CREB in same PVDF membrane. All experiments were performed in m-plicate

**
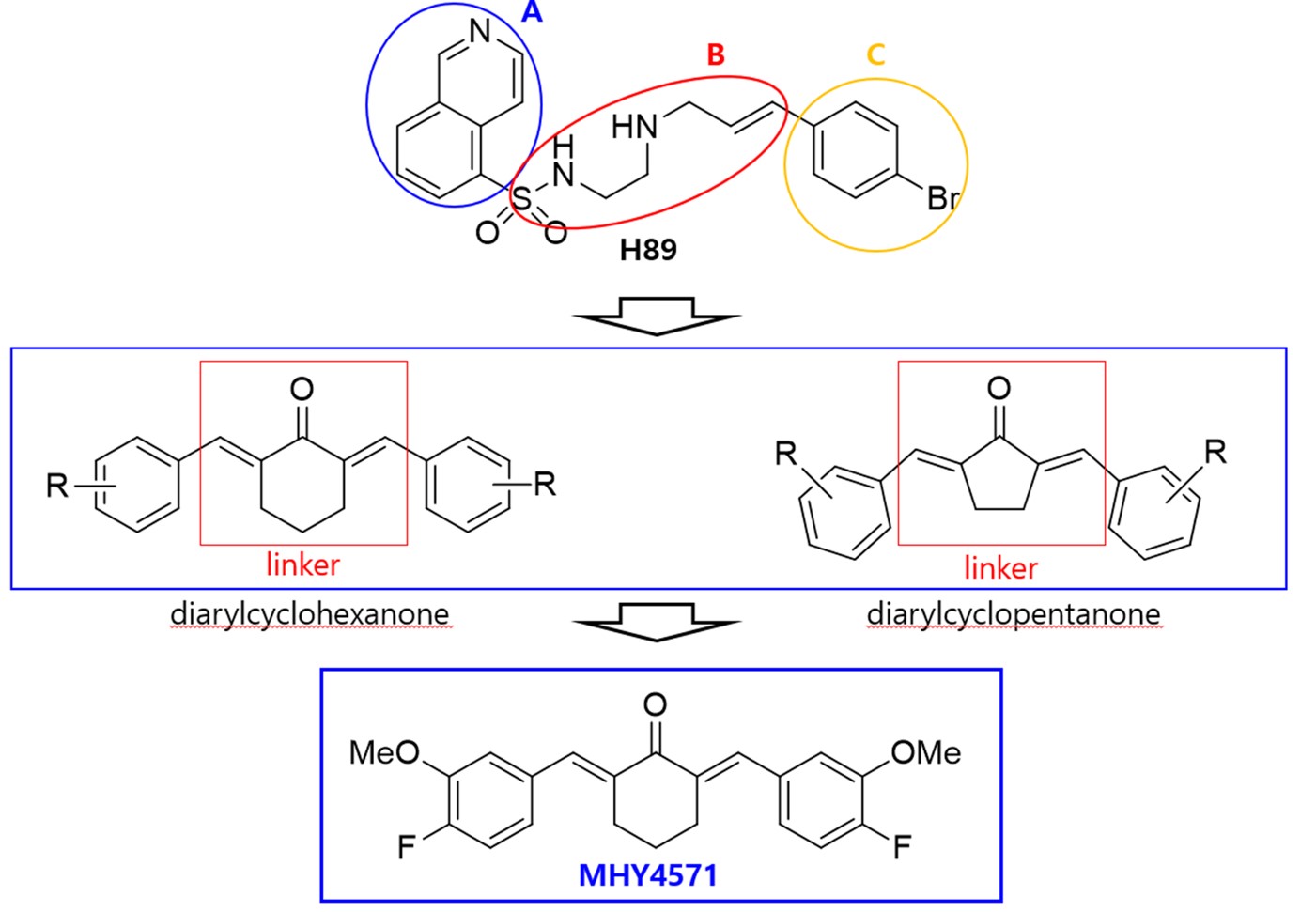
**

**Supplementary Fig. 2. Design of a novel PKA inhibitor, MHY4571, from the structure of the well-known PKA inhibitor H89.** In compound H89, the A and C moieties represent the aryl moiety, and the B moiety represents the linker.


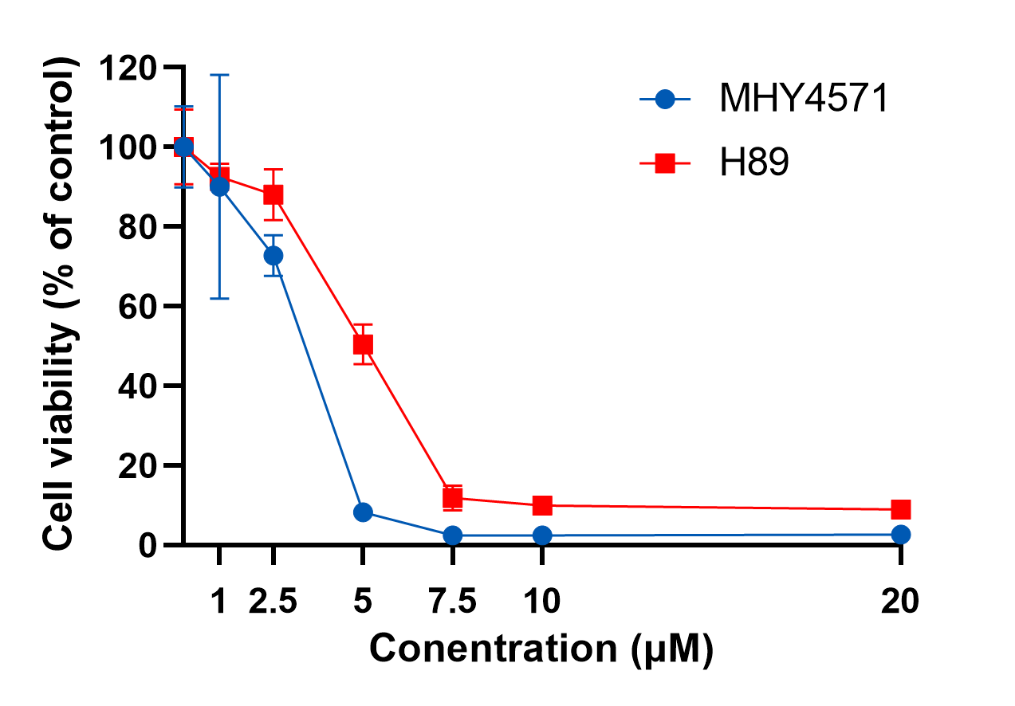


**Supplementary Fig. 3. Cytotoxic effects of MHY4571 and H89 treatment in H1703 cell lines.** Graph shows the viability of cells treated with MHY4571 or H89 for 72 hr in H1703 cell lines. All experiments were performed in triplicate, and data are expressed as the mean ± standard deviation.


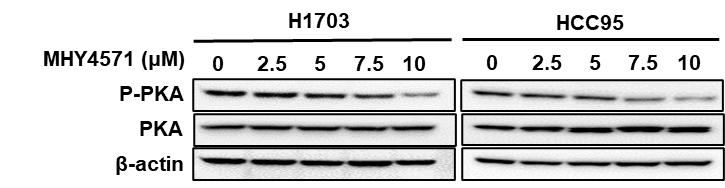


**Supplementary Fig. 4. PKA and phosphor-PKA levels in H1703 and HCC95 cells after MHY4571 treatment.** Western blot analysis showed that p-PKA levels tended to decrease while PKA levels retained no changes. All experiments were performed in triplicate


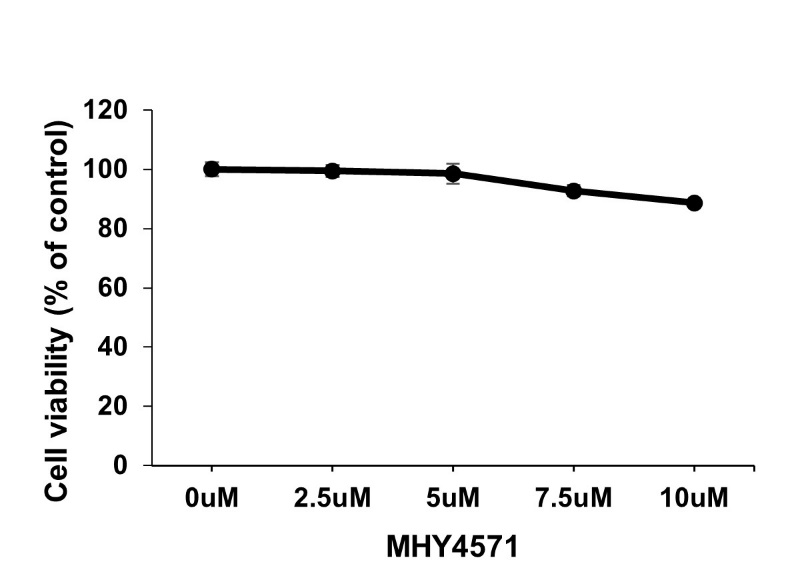


**Supplementary Fig. 5. Effect of MHY4571 on cell viability of LC-2/ad cells.** Graph showing the viability of cells treated for 24 hours with increasing concentrations of MHY4571. Results are mean ± standard deviation, n=3, expressed as a percentage of vehicle-treated control.

**Supplementary Table S1. Key resources table**

| **Resources or Reagent** | **Sources** | **Identifier** |
| --- | --- | --- |
| **Antibodies** |  |  |
| P-PKACα (Thr197) | Cell signaling | 4781 |
| PKACα | Cell signaling | 4782 |
| PARP | Cell signaling | 9542 |
| Procaspase-3 | Cell signaling | 9662 |
| P-AKT (Ser473) | Cell signaling | 9275 |
| AKT | Cell signaling | 9272 |
| p-p44/42 MAPK (Erk1/2) | Cell signaling | 9101 |
| p44/22 MAPK (Erk1/2) | Cell signaling | 9102 |
| P-CREB (Ser133) | Abcam | ab32096 |
| CREB | Abcam | ab32515 |
| E2F1 | Santa cruz | sc-251 |
| E2F2 | Abcam | ab138515 |
| E2F8 | Genetex | GTX55599 |
| β-actin | Bethyl | A300-491A |
| (HRP)-conjugated secondary antibodies | Enzo Life Sciences | ADI-SAB-300/ADI-SAB-100 |
| **siRNA** |  |  |
| PKA siRNA | Bioneer |  |
| CREB siRNA | IDT&Mbiotech |  |
| **Chemicals, Peptides, and Recombinant Proteins** | | |
| Cisplatin | Selleckchem | S1166 |
| Crystal violet | Sigma-Aldrich | V5265 |
| Bronchial Epithelial Cell Growth Medium Bullet Kit | Lonza | CC-3170 |
| RPMI 1640 | Welgene | LM 011-01 |
| HAMS F12 | Welgene | LM 010-02 |
| FBS | Welgene | S001-01 |
| Opti-MEM | Gibco | 31585-070 |
| Lipofectamine RNAiMAX | Invitrogen | 13778-075 |
| TRIzol | Sigma-Aldrich | T9424 |
| DAB staining | Dako | K5007 |
| **Critical Commercial Assays** |  |  |
| FITC Annexin V Apoptosis Detection kit | BD Biosciences | 556547 |
| cDNA synthesis platinum master mix | GenDEPOT | R5600 |
| 2X Real-Time PCR Smart mix | SolGent | SRH71-M40h |
| PKA Kinase Activity Assay | Enzo Life Sciences | ADI-EKS-390A |

**Supplementary Table S2. Primer sequence**

| **Primer ID** | **Sequences** |
| --- | --- |
| PRKACA_F | ATAAATACCTCGGCTGGCGT |
| PRKACA_R | TTCCCGGTCTCCTTGTGTTT |
| CREB_F | GACCACTGATGGACAGCAGATC |
| CREB_R | CAGGATGCCATAACAACTCCAGG |
| E2F1_F | CCATCAGTACCTGGCCGAGAGC |
| E2F1_R | CGCTTCTGCACCTTCAGCACCT |
| E2F2_F | GGCCAAGAACAACATCCAGT |
| E2F2_R | TGTCCTCAGTCAGGTGCTTG |
| E2F8_F | CCACCACAGCAAATATCGTG |
| E2F8_R | CTTTGGCCTCAGGTAATCCA |

**Supplementary Table S3. In silico ADME for MHY4571**. PreADMET (ver2.0; <https://preadmet.webservice.bmdrc.org/>) was used to determine ADME prediction for MHY4571.

**Supplementary Table S4. The kinase inhibition profile of MHY4571.** The Selectivity was determined by measuring residual activity values at one concentration in duplicate (see in 410 kinase assays (397 protein kinase assays and 13 lipid kinase assays). (ref. Excel file)
